# Bioswitches: Towards programmable, on‐demand control of therapeutic proteins

**DOI:** 10.1002/ctm2.70612

**Published:** 2026-02-02

**Authors:** Benedict Wolf, Jan Mathony, Dominik Niopek

**Affiliations:** ^1^ Institute of Pharmacy and Molecular Biotechnology (IPMB), Faculty of Engineering Sciences Heidelberg University Heidelberg Germany

1

Modern biomedicine increasingly relies on proteins as functional agents such as engineered antibodies, cytokines, or CRISPR genome editors. Most of these molecules act continuously as long as they are present in the body. This can pose challenges, particularly when pharmacokinetics is long‐lived. Adverse effects such as off‐target activity and toxicity may arise in consequence of prolonged drug activity and are difficult to mitigate once a patient has received the therapeutic. This is most extreme in gene‐based approaches, where therapeutic proteins are often continuously expressed in engineered cells or in vivo. A prominent example is CAR‐T cell therapy, in which patient‐derived T cells are modified to express tumour‐targeting receptors and can persist for months, or even years, after infusion. Similarly, in vivo gene supplementation therapy commonly relies on long‐term viral vector‐mediated expression, often intended for a lifetime. These modalities would strongly benefit from the ability to control effector protein activity post‐delivery, enabling dose adjustment and rapid risk mitigation if adverse effects should arise.

A compelling solution is to convert proteins into controllable nano‐devices, which we here term ***Bioswitches***. In our definition, a Bioswitch is an effector protein functionally coupled to a fused sensory domain, enabling reversible ON/OFF control by a defined stimulus such as small molecules, light, or changes in temperature (Figure 1A). This control mechanism relies on allostery, where protein activity is regulated by a sensory module detecting the presence or absence of a stimulus. This induces a local conformational change which is then transmitted, sometimes over long distances, to the protein's functional site, thereby modulating activity (Figure 1A). Owing to its central role in natural proteins, Jacques Monod famously referred to allostery as the “second secret of life”.

**FIGURE 1:**
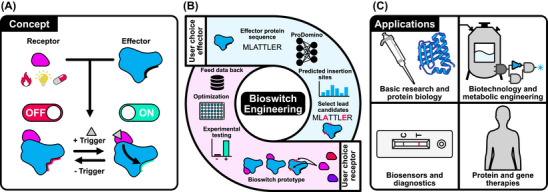
Concept, engineering and application of Bioswitches for stimulus‐dependent control of protein function in living cells and organisms. (A) Bioswitches are created through genetic fusion of sensory domains (receptor) into allosteric surface sites of effector proteins, resulting in stimulus‐dependent activity control. (B) Workflow for Bioswitch engineering. First, the ProDomino model is applied to predict suitable fusion sites in an effector protein of choice. Sensory domains that generate an allosteric signal in response to a user‐defined stimulus are then inserted into these sites, followed by experimental validation and, if required, optimization. (C) Opportunities for Bioswitch applications.

To build Bioswitches based on allostery, domain insertion engineering is commonly applied. In short, sensory domains are artificially transplanted into effector proteins via genetic fusion. This approach, however, has been bottlenecked by the challenge of identifying domain insertion sites in effector proteins suitable for sensor fusion, that is sites that preserve function while enabling strong stimulus‐dependent regulation through allosteric coupling.

In Wolf et al.^1^, we introduced ProDomino (Protein Domain Insertion Optimizer), a machine‐learning pipeline that predicts possible domain insertion sites in custom effector proteins to enable rapid creation of Bioswitches. ProDomino was trained on a large protein sequence dataset that captures natural domain‐domain coupling. Specifically, the dataset was built from proteins in which evolution has “nested” one domain within another, a phenomenon that implies structural compatibility and interdomain communication. Operationally, ProDomino takes a custom effector protein sequence as input and outputs a position‐resolved insertion‐likelihood profile, highlighting sites suitable for fusion of a sensory domain.^1^ Hence, ProDomino accelerates Bioswitch creation: Users define: (i) the effector and sensory domain (control modality), (ii) employ ProDomino to “make the match” between them, and (iii) perform experimental validation and, if required, optimization of Bioswitch designs (Figure 1B).

In our study, we employed this pipeline to create Bioswitches based on the *E. coli* transcription factor AraC, CRISPR‐Cas effectors relevant to gene therapies, and multiple enzymes—without requiring time consuming and costly experimental screening.^1^ Importantly, this pipeline is modular with respect to the activating stimulus: sensory domains responsive to light, clinically approved drugs, and temperature changes can all be integrated^1^, ^2^ and recent progress in receptor‐domain engineering increasingly broadens the input choice.^2^, ^3^, ^4^, ^5^, ^6^ Collectively, ProDomino aims to shift domain insertion engineering from a major experimental undertaking towards one‐shot, design‐driven construction of Bioswitches.

Thereby, ProDomino‐informed Bioswitches empower basic research and biotechnology, and open ample opportunities in the diagnostics and therapeutics space (Figure 1C). Starting with the most immediate impact, Bioswitches already provide a powerful route to interrogating dynamic processes in biological systems. By matching perturbations to biological timescales and spatial organization of cells and organisms, Bioswitches are, since many years, employed for the dissection of signalling networks, gene regulation, and cell‐fate decisions. In this context, optogenetic Bioswitches, employing light‐sensing photoreceptors, offer second‐to‐minute resolution and spatial precision. Likewise, chemogenetic Bioswitches use ligand‐binding domains and facilitate deep‐level control through small molecules. Beyond being employed as probing tools, Bioswitches also act as interesting study objects of protein biology itself, because successful designs reveal where proteins tolerate structural perturbation and how conformational changes propagate.

Another natural application area of Bioswitches is Biotechnology and specifically metabolic engineering. In this context, controllable enzymes can serve as adjustable nodes in metabolic cascades to dynamically balance flux, decouple growth from production, and manage pathway toxicity, thus improving grade and yield in bioproduction processes.

Currently, Bioswitches also gain momentum in the space of biosensing and diagnostics. The coupling of molecule recognition to robust enzymatic or fluorescent outputs enables practical assay formats for biomarker detection, drug monitoring, or point‐of‐care readouts. Various successful examples of biosensors that follow the Bioswitch paradigm have been reported,^7^, ^8^ thus far mostly built via trial‐and‐error engineering, a process that ProDomino can now help streamline and accelerate.

The most transformative—and also most challenging—opportunity lies in controllable protein therapeutics, where Bioswitches could convert potent yet high‐risk modalities into doseable, reversible precision interventions. Antibodies, cytokines, and other effectors could be engineered to toggle activity in response to clinically feasible inputs, allowing post‐delivery modulation of function when adverse effects emerge or when therapeutic windows shift over time. In addition, spatially confined Bioswitch stimulation (e.g., light, focused ultrasound, localized heating/cooling, or magnetic fields) could, in principle, bias activity towards the intended tissue and reduce systemic off‐target effects. A parallel rationale applies to gene therapy: encoding controllable Bioswitches rather than permanently active transgenes could introduce an adjustable layer atop durable expression, improving safety and flexibility for long‐lived interventions.

Realizing these ambitions will require addressing several conceptual and practical challenges to translate proof‐of‐concept bioswitch design into clinical‐grade performance. This will involve further optimizing the ProDomino pipeline towards robust applicability across protein families, tightening receptor‐effector coupling to achieve near digital performance and/or wide‐range tunability, for example through directed evolution strategies,^8^, ^9^ addressing the issue of potential immunogenicity of designer Bioswitches as well as enabling their scalable production and quality control. Tackling these challenges promises innovations ranging from basic research, through biotechnology to diagnostics and future therapies.

## CONFLICT OF INTEREST STATEMENT

The authors are inventors of already filed and/or soon‐to‐be filed patent applications related to the Bioswitches concept.
